# Size dependence of the surface spin disorder and surface anisotropy constant in ferrite nanoparticles†

**DOI:** 10.1039/d3na00266g

**Published:** 2023-08-03

**Authors:** Marianna Gerina, Marco Sanna Angotzi, Valentina Mameli, Veronika Gajdošová, Daniel N. Rainer, Milan Dopita, Nina-Juliane Steinke, David Aurélio, Jana Vejpravová, Dominika Zákutná

**Affiliations:** a Department of Inorganic Chemistry, Faculty of Science, Charles University Hlavova 2030/8 128 43 Prague 2 Czech Republic zakutnad@natur.cuni.cz; b Department of Chemical and Geological Sciences, University of Cagliari S.S. 554 bivio per Sestu, 09042 8 Monserrato CA Italy; c Institute of Macromolecular Chemistry, Academy of Sciences of the Czech Republic 162 06 Prague 6 Czech Republic; d Department of Physical and Macromolecular Chemistry, Faculty of Science, Charles University Hlavova 2030/8 128 43 Prague 2 Czech Republic; e Department of Condensed Matter Physics, Faculty of Mathematics and Physics, Charles University Ke Karlovu 5, 121 16 Prague 2 Czech Republic; f Institut Laue-Langevin 71 Avenue des Martyrs F-38042 Grenoble France

## Abstract

The magnetic properties of nanoscale magnets are greatly influenced by surface anisotropy. So far, its quantification is based on the examination of the blocking temperature shift within a series of nanoparticles of varying sizes. In this scenario, the surface anisotropy is assumed to be a particle size-independent quantity. However, there is no solid experimental proof to support this simplified picture. On the contrary, our work unravels the size-dependent magnetic morphology and surface anisotropy in highly uniform magnetic nanoparticles using small-angle polarized neutron scattering. We observed that the surface anisotropy constant does not depend on the nanoparticle's size in the range of 3–9 nm. Furthermore, our results demonstrate that the surface spins are less prone to polarization with increasing nanoparticle size. Our study thus proves the size dependence of the surface spin disorder and the surface anisotropy constant in fine nanomagnets. These findings open new routes in materials based on a controlled surface spin disorder, which is essential for future applications of nanomagnets in biomedicine and magnonics.

## Abstract 2

Size dependence of the total magnetized volume of nanoparticles in a magnetic field.

## Graphical abstract

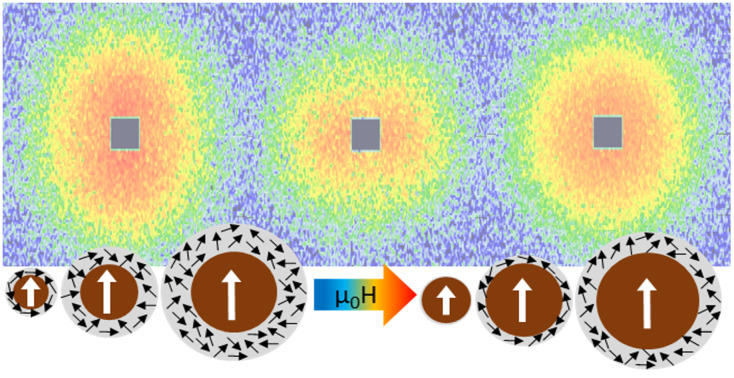

## 1 Introduction

Disorder, the most favorable phenomenon for reducing a system's total energy, crucially alters materials' physical and chemical properties, whether in the form of chemical, structural, magnetic, or geometric disorder.^1,2^ Therefore, in material science, controlling and tuning disorders is beneficial. Decreasing the size of materials to the nanoscale, the effect of disorder becomes increasingly important.^3^ Magnetic nanoparticles (NPs) promising technological and biomedical applications in for example,^4,5^ magnetic recording,^6–10^ magnetic fluid hyperthermia,^11–13^ magnetic manipulation for isolation of target biomolecules and rapid mixing,^14,15^ and magnetic resonance imaging (MRI)^16,17^ are due to their particular physical properties arising from their large surface-to-volume ratio. Different applications require distinct magnetic properties, for instance, data-storage applications demand high magnetic anisotropy to preserve thermal stability,^18^ while magnetic hyperthermia and MRI necessitate superparamagnetic NPs.^19^ The resulting magnetic properties and performance are affected not only by the disorder but also by various aspects, such as shape,^20–22^ size,^23–26^ chemical composition,^27^ crystal phase,^28^ surface coating^29^ and effects.^30,31^ However, surface disorder phenomena that arise from vacancies or the presence of antiphase boundaries,^32–36^ such as spin disorder^37^ and spin canting,^38,39^ have the strongest influence on the magnetic properties of NPs,^3^*i.e.*, spontaneous magnetization, superparamagnetic behavior, coercivity, and exchange interaction. The effective magnetic anisotropy energy, *E*_a_ = *K*_eff_·*V*, describes the alignment of the particle's moment in the field direction, and it is an important parameter for many applications, for example, in data storage, it describes the efficiency of storing medium. *E*_a_ depends on the effective anisotropy constant, *K*_eff_, and volume of material, *V*, and thus, studying their correlation with the particle size is important.^40^ In small NPs, where a large surface-to-volume ratio drives the magnetic properties, the effective anisotropy constant depends not only on the bulk magnetocrystalline anisotropy, *K*_b_ but also on the contribution from surface atoms. For spherical NPs, the effective anisotropy constant can be described as follows:

1 *K*_eff_ = *K*_b_ + 6/*d*·*K*_s_,

where *d* and *K*_s_ represent the particle diameter and surface anisotropy constants, respectively, and 6/*d* is the ratio of the surface area to the volume of a spherical nanoparticle.^41,42^ Until now, the *K*_eff_ was extracted from the shift of the blocking temperature using two different approaches, either from the real part of the susceptibility (AC susceptometry) or from IRM experiments,^43–49^ and the obtained *K*_s_ was attributed to the material. However, the assumption that the surface anisotropy constant is size-independent for samples prepared with the same synthesis method has never been proven experimentally. In contrast, magnetic small-angle polarized neutron scattering (SANS) studies of magnetic NPs uncovered that spin textures of NPs can be complicated, having uniform and different non-uniform, canted, or core–shell-type configurations.^50–63^ Recently, a study by Zákutná *et al.* spatially resolved for the first time the surface disorder energy within one NP-size batch using half-polarized SANS (SANSPOL).^64^ The disorder energy, *E*_dis_ is defined as

2 *E*_dis_ = *μ*·*H*·*M*_z_(*H*)·[*V*_mag_(*H*) − *V*_mag_(*H*_min_)],

where *M*_z_(*H*), *V*^*H*^_mag_^_max_^, and *V*_mag_(*H*_min_) are the longitudinal magnetization at the applied field, and magnetized volumes at *H*_max_ and *H*_min_, respectively. Afterward, they accessed the effective anisotropy constant according to the following equation:3
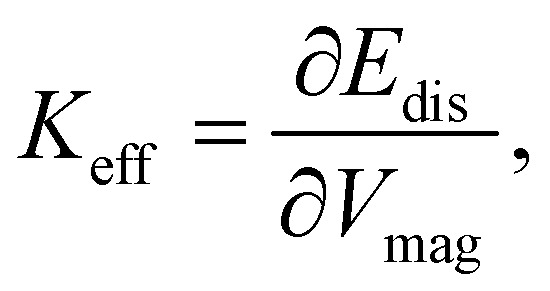
where ∂*E*_dis_ and ∂*V*_mag_ are the derivatives of the disorder energy and magnetic volume, respectively. From the effective anisotropy constant, the spatially resolved surface anisotropy constant, *K*_S_, can be obtained:4
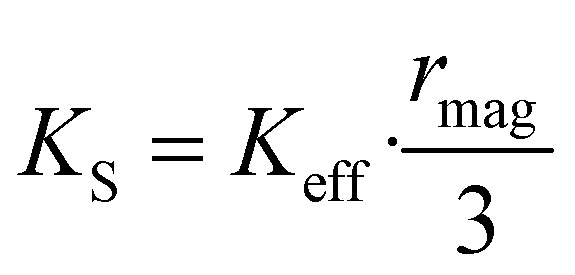


Thus, with the aim to validate experimentally eqn (1), according to which the surface anisotropy constant does not depend on the size of the material, using SANSPOL experiments and the analysis proposed by Zákutná *et al.*,^64^ we studied the surface spin disorder in spherical CoFe_2_O_4_ NPs, prepared with the solvothermal method, having different coherent domain sizes of 3.1(1), 6.3(1), and 8.6(1) nm and narrow size distribution of *σ*_log_ < 20%. To isolate the effect of the size, we selected samples having the same chemical composition, crystalline and particle shape, and without interparticle interactions. For the first time, we prove that albeit the energy needed for the polarization of the spins into the field direction dramatically changes with NP size, the surface anisotropy constant remains the same in this size range. Moreover, we show that even applying the saturation field, not all the spins at the NP surface are polarized along the field direction, and the degree of polarization depends on the total crystalline and NP size. This study addresses, for the first time experimentally, open fundamental questions about the surface anisotropy constant and progresses the design and characterization of magnetic NPs for their future technological and biomedical applications.

## 2 Experimental

### 2.1 Nanoparticle synthesis

#### 2.1.1 Chemicals

Oleic acid (>99.99%), 1-pentanol (99.89%), hexane (84.67%), and toluene (99.26%) were purchased from Lach-Ner; 1-octanol (>99.99%) from SigmaAldrich; absolute ethanol and Co(NO_3_)_2_·6H_2_O (99.0%) from Penta; NaOH (>98.0%) from Fluka; Fe(NO_3_)_3_·9H_2_O (98.0%) from Lachema.

#### 2.1.2 Synthesis

The nanoparticles were synthesized by the hydrolysis of the Co(ii) and Fe(iii) oleates under solvothermal conditions, as reported by Sanna Angotzi *et al*.^65^ The metal-oleates were prepared according to Repko *et al*.^66^ An aqueous solution of Co(ii) and Fe(iii) nitrates in a ratio of 1 : 2 was added to a solution of sodium oleate, obtained by mixing sodium hydroxide with oleic acid in water and ethanol mixture (1 : 1). Afterwards, 20 mL of hexane was added, and the mixture was kept at the reflux temperature for 1 h. Then, the reaction solution was cooled down to room temperature. The water phase was removed, and the organic phase was mixed with 20 mL of water, 5 mL of ethanol, and 5 mL of hexane and refluxed for another 30 min to remove the inorganic residuals. This step was repeated twice. Finally, the water phase was discarded from the mixture, and the remaining organic solvents were evaporated. The viscous metal-oleate product was then dissolved in 15 mL of pentanol. Cobalt ferrite NPs were synthesized by a solvothermal method of the metal-oleate complex at 220 or 180 °C for 10 h in a stainless-steel autoclave (Berghof DAB-2). The details of the synthesis are reported in the Table S1.† After synthesis, the NPs were first separated from the solvent with a permanent magnet. Then, NPs were washed twice using 10 mL of hexane and 10 mL of water in ultrasound bath with a magnet separation to remove the residue of oleic acid and organic solvents. Afterward, the NPs were dispersed in 5 mL of hexane and centrifuged at 3000 rpm for 5 min to remove agglomerates. Only the stable part of the NP dispersion was kept for further characterization. The sample names are labelled as S3, S6, and S9 sample, where the number indicates coherent domain size and letter S the spherical morphology.

### 2.2 Electron microscopy

Transmission electron microscopy (TEM) measurements were done at the Tecnai G2 Spirit microscope from FEI operating at 120 kV, and high-resolution scanning TEM (HRSTEM) measurements were carried out on JEOL NEOARM 200 F operating at 200 kV equipped with Schottky-FEG cathode and C_S_ corrector, respectively. In both techniques, the toluene dispersion of NPs was dropped at the Cu grid with 400 mesh coated by carbon foil. The TEM micrographs were taken in bright-field mode, and the size distribution was made by manually measuring at least 200 particle sizes from different TEM micrographs in ImageJ software.^67^ Acquisition of HRSTEM micrographs was made in annular bright (ABF) and dark-field (ADF) mode.

### 2.3 Powder X-ray diffraction

Powder X-ray diffraction (PXRD) measurements were performed at the Panalytical X'pert Pro diffractometer equipped with Cu K_α_ radiation and a secondary monochromator. The data were measured in powder form at the glass holder. The data analysis was done by Rietveld refinement with implemented spherical harmonics function in the WinPLOTR software within the FullProf software package.^68^ The instrumental broadening contribution of the diffractometer was extracted from the standard measurement of LaB_6_ from NIST. Within the Rietveld analysis, the spherical harmonics function^69^ describing the preferred orientation of crystallites was used to obtain averaged crystallite shape. The coherent domain size and shape were visualized by the GFourier program within the FullProf.^68^

### 2.4 Small-angle X-ray scattering

Small-angle X-ray scattering (SAXS) experiments were performed at Xenocs Xeus 2.0 equipped with Cu and Mo K_α_ microfocus X-ray sources, toroidal parallel beam producing X-ray mirrors, two sets of beam collimating scatter-less slits and Dectris PILATUS 200K detector. The experiments were performed at a detector distance of 2.50 m using both Cu and Mo wavelengths to cover the maximum accessible *Q*-range. Measured 2D intensity profiles were azimuthally integrated. Resulted 1D SAXS patterns were corrected to the capillary/sample thickness, the transmission of samples, and properly scaled solvent and capillary signal was subtracted.

### 2.5 Small-angle neutron scattering

Half-polarized small-angle neutron scattering (SANSPOL) was carried out at the D33 instrument at the Institut Laue-Langevin, Grenoble, France.^70^ The experiments were done in two instrument configurations with 2.8 and 7.8 m collimation and 2 and 7.8 m detector to sample distance, respectively. Due to the small size of the S3 sample, SANSPOL data were collected only at high *Q*-values. The neutron beam of the wavelength of 5 Å with the wavelength spread of Δ*λ*/*λ* = 10% was used. The aperture of the rectangular shape and dimension of 5 × 7 mm was used to focus the neutron beam on the sample. At the sample, the magnetic field up to 1.34 T was applied (other applied magnetic fields: 0.7, 0.1, 0.05, 0.03, 0.02, and 0.01 T) in the horizontal direction perpendicular to the neutron beam. The data were corrected to the background scattering, blocked beam, and polarization efficiency of the V-shaped polarizer (0.91) and flipper (0.99). The scattering intensity was transformed to the absolute scale by measuring the intensity of the empty beam. The pure nuclear scattering cross-section was extracted by sector averaging (with a total opening of 20°) in the magnetic field direction (horizontal) at the saturating magnetic field (1.34 T). The nuclear-magnetic scattering cross sections were obtained from sector averaging (with a total opening of 20°) perpendicular to the applied magnetic field (vertical) for all used external magnetic fields.

### 2.6 Macroscopic magnetization

Magnetization measurements were performed in a physical property measurement system (PPMS) from Quantum Design (QD) equipped with Vibrating Sample Magnetometry (VSM) module. The measurements were done on powder samples enclosed in capsules with epoxy glue to prevent the physical rotation of the grains in the magnetic field. Temperature dependence of magnetization was measured under zero-field cooled (ZFC), and field-cooled conditions to 2 K, and the magnetization response of the samples were collected while warming the samples from 2 K to 350 K with an applied field of 50 mT. The isothermal magnetizations were collected at several temperatures (10 K, 50 K, 60 K, 100 K, 298 K, and 350 K depending on the sample) in the range of the applied magnetic field of ±7 T.

### 2.7 Micromagnetic simulations

The micromagnetic simulations were performed using mumax3.^71^ The simulations were based on single NPs of cubic computational cells of 1 nm, both uniaxial and cubic anisotropies were considered in separate simulations.

### 2.8 Thermogravimetric analysis

Thermogravimetric analysis (TGA) was carried out at the was performed on SETARAM SETSYS evolution 1750 in the nitrogen atmosphere (40 mL min^−1^). Powder samples (3–10 mg) were placed into a 100 μL alumina crucible and mounted on a Pt/Rh DSC rod. Samples were heated up to 800 °C with a heating rate of 5 °C min^−1^.

### 2.9 Inductively coupled plasma optical emission spectroscopy

Inductively Coupled Plasma-Optical Emission Spectrometry (ICP-OES) was performed using an Agilent 5110 device. The calibration line was performed in the range 1–10 mgL^−1^ at wavelengths 228.615 nm for cobalt and 238.20 nm for iron. The samples were prepared for the analysis as follows: 1 mL of H_2_O_2_ and milliQ water were added to approximately 6 mg of the powder sample under magnetic stirring to decompose the organic shell. When the formation of the foam was not observed anymore, we added 6 mL of HCl and 2 mL of HNO_3_. Then, the solution was heated at 80 °C for 2 h and filtered in a volumetric flask.

## 3 Results and discussion

### 3.1 Structure, morphology, and chemical composition

**Fig. 1:**
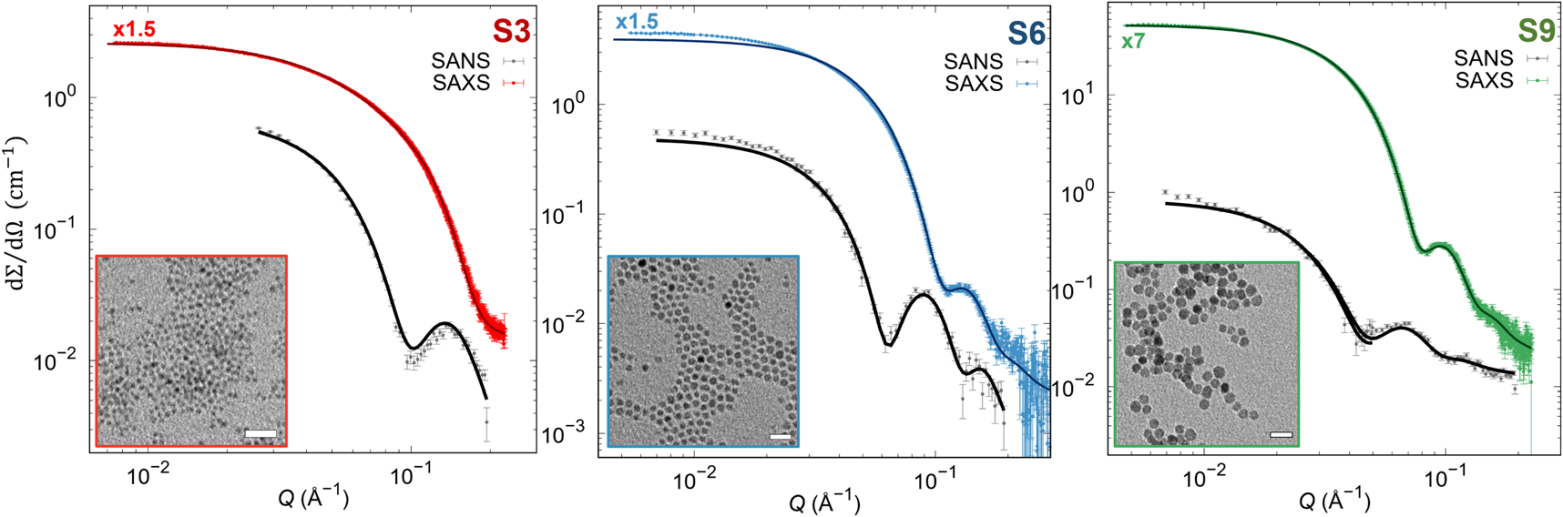
SAXS and pure nuclear SANS cross-section (points) with form factor refinements (full lines). The SAXS data were scaled by 1.5 or 7-times as indicated in the graphs above the SAXS data. (Insets) TEM micrographs in bright field mode. Scale bars: 100 nm.

To reveal precisely the size dependence of the surface anisotropy constant, differently sized cobalt ferrite NPs of the same shape and reasonable size distribution were synthesized with the same protocol, according to Sanna *et al*.^65^ The precise stoichiometry of Co_0.96_Fe_2.03_O_4_ was confirmed by inductively coupled plasma optical emission spectroscopy for all samples. Furthermore, all samples consist of spherically shaped NPs with different mean particle sizes 4.4(1) (S3 sample), 8.1(1) (S6 sample), and 11.1(1) nm (S9 sample) having a reasonably narrow size distribution of *σ*_log_ < 20% determined from SAXS (Table S2†), which is in good agreement with the TEM results (Fig. S2 and Table S2†). Moreover, using the Rietveld analysis with implemented spherical harmonic function, we obtained a spherical shape of the coherent domain size for all the samples, as reported in the inserts in Fig. S1 and Table S2.† The Rietveld analysis revealed coherent domain sizes of 3.1(1), 6.3(1), and 8.6(1) nm for S3, S6, and S9 samples, respectively, which are significantly smaller than their physical particle size. This disagreement between crystallite size and particle size is due to the structural disorder or lack of crystallinity at the NP's surface confirmed also by HRSTEM (Fig. S3†), leading to a possible presence of surface effects. It must be stressed that the exact stoichiometry, narrow size distribution, and crystalline and physical shape make the set of samples suitable for studying merely the effect of the physical size on the surface anisotropy constant. Furthermore, we extracted pure nuclear scattering cross-sections at the saturation field of the sample from SANSPOL experiments to retrieve additional information on the NP's nuclear morphology. The pure nuclear scattering cross-sections (Fig. 1) were described using the core-surfactant model, wherein the core represents the inorganic particle size, and the surfactant corresponds to the oleate (OA) ligand shell. The core particle sizes were fixed according to the obtained SAXS results (*r*_nuc_), and only the OA shell thickness (*d*_OA_) was refined (with fixed *ρ*_OA_ = 0.78 × 10^−7^ Å^−2^), leading to the thickness of *d*_OA_ = 1.3(1) nm for all samples (as reported in Table S2†), in agreement with previously published results.^64,72^ Moreover, the presence of the Guinier plateau at the low *Q*-ranges in nuclear SANS cross-sections indicates that the dispersions consist of non-interacting NPs.

### 3.2 Averaged macroscopic magnetic properties

From zero-field-cooled and field-cooled curves of samples (Fig. S4†), the typical shift of the blocking temperature to higher values with increasing the physical size of NPs is obtained (see further description in the ESI, Table S6†). From isothermal macroscopic averaged magnetization measurements, bimodal magnetic dipole distribution was obtained by numerical inversion method (Fig. S5†), supporting the possible presence of spin disorder but also interparticle interactions that are commonly present in powder samples. However, one broad monomodal magnetic moment distribution can describe these bi-modal distributions. The averaged dipole magnetic moment of 3.35 × 10−^21^ A m^−1^ (361 μ_B_), 8.70 × 10−^21^ A m^−1^ (938 μ_B_), and 1.60 × 10−^20^ A m^−1^ (1730 μ_B_) was obtained for S3, S6, and S9 sample, respectively. It has to be mentioned that samples were measured in powder form, and thus results might be influenced by interparticle interactions as well as spin disorder effects. Moreover, to support our experimental observations, we performed micromagnetic simulations using mumax3 (Fig. S6–S8†).^71^

### 3.3 Effective and surface anisotropy

**Fig. 2:**
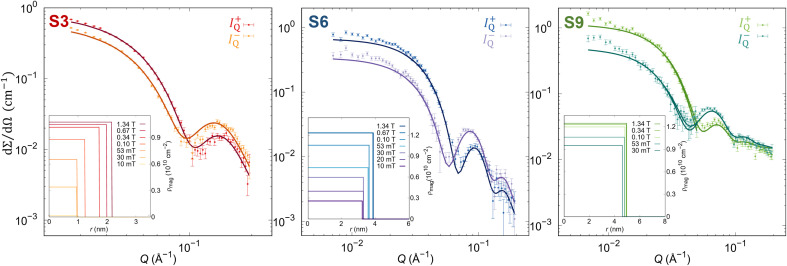
SANSPOL scattering cross sections (points) for the polarization *I*^−^_Q_ and *I*^+^_Q_ with core–shell-dead layer form factor refinements (full lines) at 1.34 T. (Insets) Obtained radial distribution of *ρ*_mag_ at different applied magnetic fields.

To unveil surface phenomena in the prepared set of samples, the magnetic morphology of single NP has to be resolved with the spatial resolution. Therefore, magnetic SANS experiments were done on the NPs dispersion with low concentrations (1–3 mg mL^−1^) to avoid the effects of the interparticle interaction on the NP's magnetic properties.^73^ The magnetic morphology of the samples was revealed by the core–shell-surfactant form factor refinement of the vertical sectors (90° to the applied field with 20° opening) of the magnetic-nuclear cross-sections (*I*^−^_Q_, *I*^+^_Q_) at different applied magnetic field strengths (Fig. 2, Table S3–S5 in the ESI†).

In this model, we define the magnetized part of the NPs as the core (*r*_mag_), while the shell is the non-magnetized part, named the disordered layer (*d*_dis_), which allows us to unveil the presence of possible surface phenomena. At the maximal applied magnetic field (*H*_max_ = 1.34 T), all samples show a smaller magnetic radius than the physical size, confirming the presence of the layer without magnetization. Nevertheless, the amount of the magnetized volume decreases with increasing NP size. The smallest NPs (S3 sample) are almost fully magnetized with only 7.6(2)% of non-magnetized part, while for larger particles, the disordered volume fraction increases. Indeed, the largest NPs (S9 sample) have a non-magnetic volume of 25.9(8)% (Table 1). The magnetic scattering length density, *ρ*_mag_, increases with the applied magnetic field due to the alignment of the magnetic moment of the NPs along the field, supporting the previous work of Zákutná *et al*.^64^ Moreover, a different degree of dependence of the magnetized volume and *ρ*_mag_ with the applied magnetic field is observed for different NPs sizes (Table 1). These results report the evidence of the size dependence of the surface spin disorder.

**Table 1:** Obtained values of magnetic radius at the highest applied magnetic field (*H*_max_ = 1.34 T), *r*_mag_(*H*_max_); the thickness of a dead layer at *H*_max_, *d*_dis_(*H*_max_); magnetic scattering length density at *H*_max_, *ρ*_mag_(*H*_max_); volume fraction of spin disorder at the lowest applied magnetic field (*H*_min_ = 10 mT) *φ*_spin_(*H*_min_); volume fraction of spin disorder at *H*_max_, *φ*_spin_(*H*_max_); the magnitude of the surface spin disorder energy, Δ*E*_dis_(*H*_max_); and maximum value of effective anisotropy, *K*_eff,max_ from SANSPOL refinements

Parameter	S3	S6	S9
*r*_mag_(*H*_max_) (nm)	2.22(2)	3.87(4)	4.96(5)
*d*_dis_(*H*_max_) (nm)	0.06(2)	0.18(4)	0.52(5)
*ρ*_mag_(*H*_max_) (10^−6^ Å^−2^)	1.03(1)	1.23(3)	1.25(3)
*φ*_spin_(*H*_min_) (%)	45(22)	50(12)	40(2)
*φ*_spin_(*H*_max_) (%)	7.6(2)	13.1(4)	25.9(8)
*E*_dis_(*H*_max_) (10^−20^ J)	0.9(2)	6(2)	6(2)
*K*_eff,max_ (10^6^ J m^−3^)	8(2)	5(2)	4(1)

**Fig. 3:**
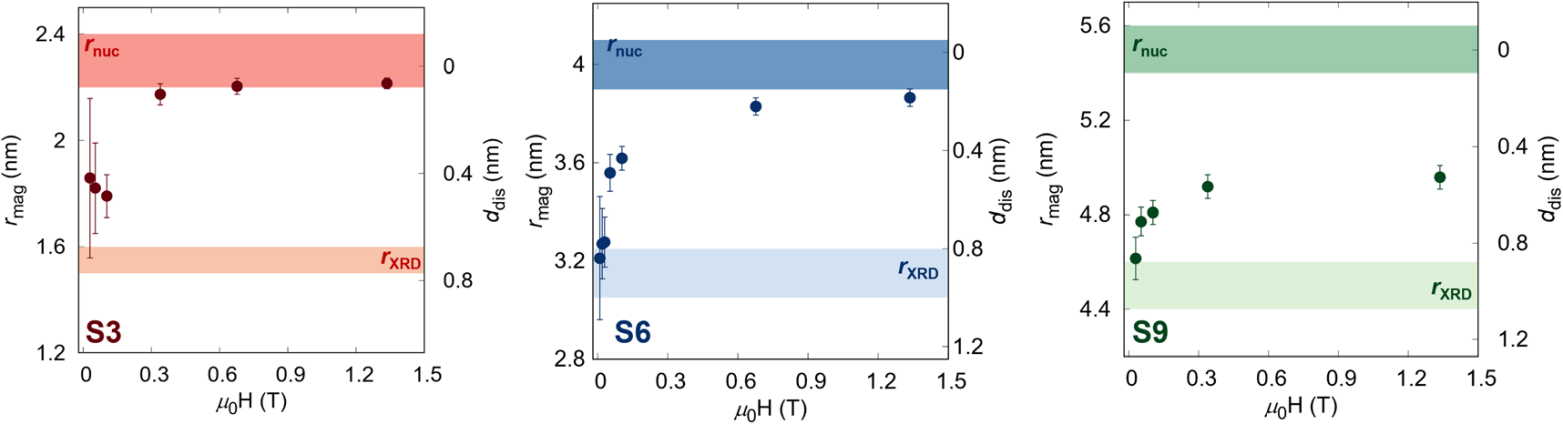
Field-dependence of the magnetic radius and the disorder layer thickness for all samples. The transparent surfaces represent the samples' crystalline size (brighter) and nuclear radius (darker).

**Fig. 4:**
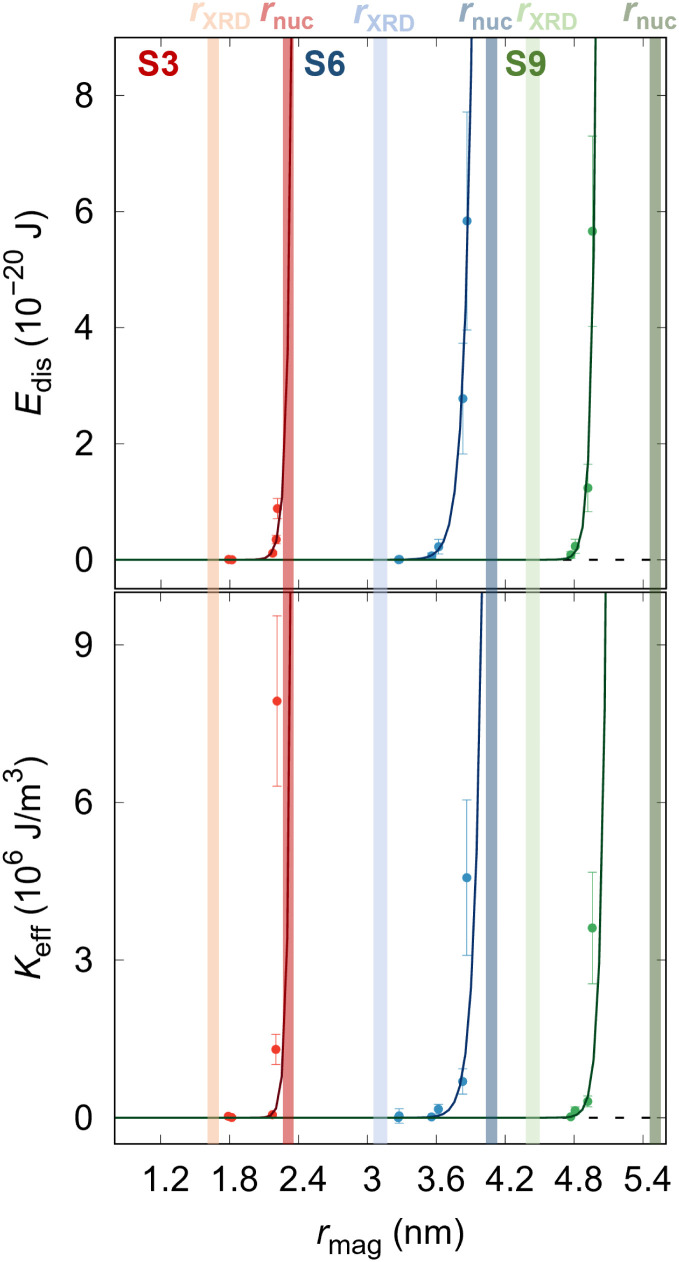
(a) Dependence of the disorder energy and (b) effective anisotropy constant on the magnetic radius of the samples. The transparent surfaces represent the samples' crystalline size (brighter) and nuclear radius (darker). Solid lines represent the guide to the eye.

The obtained field-dependence of *r*_mag_ and *d*_dis_ is visualized in Fig. 3. At the lowest applied field (*H*_min_ = 10 mT), the samples show the size of magnetized NPs close to the coherent domain size (*r*_XRD_) with a significant non-magnetic volume fraction (*φ*_spin_(*H*_min_)) between 40–50% depending on the sample (Table 1), which reduces gradually with an increasing magnetic field. Nevertheless, while the smallest sample with a coherent domain size of 3.1(1) is almost fully magnetized at *H*_max_ with only 7.6(2)%, the larger samples still have significant non-magnetic volume (up to 25.9(8)% for the S9 sample). This is understood as the smaller particles are easier to magnetize, which is directly related to the energy needed to polarize spins into the field direction, known as disorder energy. These results are in excellent agreement with macroscopic magnetization measurements, whereby the non-regularized numerical inversion approach^74^ of the magnetization curve at the 298 K, two populations of the magnetic moment were obtained corresponding to the core and shell (Fig. S5†). Moreover, the averaged magnetized radii of 2.31(1), 3.96(1), and 4.91(2) nm were extracted for S3, S6, and S9 samples, respectively, representing the same total magnetized volume as received from SANSPOL at the highest applied magnetic field. Furthermore, to obtain more in-depth information on the surface disorder, we calculated the disorder energy from SANSPOL results according to Zákutná *et al.*,^64^ using eqn (2). The longitudinal magnetization is directly proportional to the decrease of the *ρ*_mag_:5
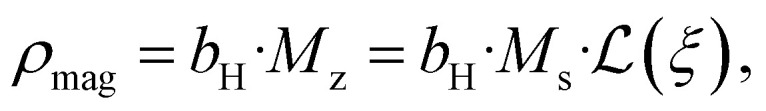
with magnetic scattering length, *b*_H_ = 2.91 × 10^8^ (Am)^−1^, spontaneous magnetization, *M*_s_, and Langevin function, 
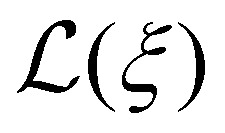
. Fig. 4 reports the field-dependence of the disorder energy (left). Comparing the trend of the *E*_dis_, we can observe that the samples S6 and S9, the magnetic size approaches the structural size in a similar way while the S3 sample differs from the other two, indicating that the energy required to polarize spins at the NP surface increases with the applied magnetic field. The obtained maximum values of *E*_dis_(*H*_max_) are reported in Table 1, where we can notice as well an increase of *E*_dis_(*H*_max_) with the NPs size, which is in line with the previous statement. From the derivative of disorder energy over magnetized volume (eqn 3), the effective anisotropy constant for all samples is accessed (Table 1 and Fig. 4b). The obtained *K*_eff_ values are in the same order of magnitude as effective anisotropy for *r*_nuc_ = 7.04 nm Co_0.22_Fe_2.52_O_4_ NPs (*K*_eff_ = 10^6^ J m^−3^).^64^ To support the phenomenological equation (eqn (1)), we spatially resolve the surface anisotropy constant according to Zákutná *et al.*,^64^*K*_S_ = *K*_eff_ × *r*_mag_/3, independently for each NP's size. As reported in Table 2, we obtained the spatially resolved *K*_S_ = 6 mJ m^−2^ for each sample, confirming that the surface anisotropy constant does not depend on the NPs size in the case of the ideal batch of samples, *i.e.* where the composition, coherent domain shape, and physical morphology is same. We also provide the volume averaged disorder anisotropy, 〈*K*_s_〉, calculated as 〈*K*_s_〉 = *E*_dis_(*H*_max_)/*V*_nuc_ × *r*_nuc_/3, by considering the whole nuclear volume of NPs from the disorder energy at the highest applied magnetic field (Table 2). Obtained 〈*K*_s_〉 values are in good agreement with previously reported values of surface anisotropies of different ferrite NPs.^46,48,75,76^ These results experimentally support the theoretical description, according to which the surface-to-volume ratio does not influence the surface anisotropy, however, the exchange interactions lead to reduced magnetic particle size compared with the structural size.^46,48,75,76^

**Table 2:** Obtained values of maximum surface anisotropy, *K*_s_, and volume averaged disorder anisotropy, 〈*K*_s_〉 from SANSPOL refinements

Parameter	S3	S6	S9
*K*_s_ (mJ m^−2^)	6(1)	6(2)	6(2)
〈*K*_s_〉 (mJ m^−2^)	0.14(1)	0.28(9)	0.15(4)

## 4 Conclusions

Within this work, we prove experimentally that the classical approach of extracting the averaged surface anisotropy constant, as interpolation of the blocking temperature shift from AC susceptometry data using different NP size batches, is entirely correct and reasonable. However, it is worth underlying that this approach should be applied only if the NPs have been prepared with the same synthesis method and possess the same chemical composition, polydispersity, and physical and crystallite shape. Furthermore, we demonstrate that NP's size increases the energy needed to polarize spins into the field direction. These observations are crucial in designing the new materials with controlled surface disorder necessary for the improved magnetic NP applications in magnetic hyperthermia, drug delivery, data storage, and the development of rare element-free permanent magnets.

## Author contributions

M. G. carried out experimental work, performed data analysis, prepared figures, and wrote the manuscript. D. Z. supervised, conceptualized, validated, wrote, and edited the manuscript. M. S. A. synthesized the S3 and S9 samples. V. M., M. G., D. Z. and N.-J. S. performed half-polarized small-angle neutron scattering experiments. V. G. carried out TEM measurements, and D. N. R. performed HRSTEM experiments. M. D. carried out SAXS experiments. D. F. C. de A. A. made micromagnetic simulations. J. V. conducted data analysis using numerical inversion. All authors have read, commented, and edited the manuscript.

## Conflicts of interest

There are no conflicts to declare.

## Supplementary Material

NA-005-D3NA00266G-s001
