# Supplementary material for: Size dependence of the surface spin disorder and surface anisotropy constant in ferrite nanoparticles
Source: Nanoscale Adv. 2023 Aug 3;5(17):4563–70. doi: 10.1039/d3na00266g (PMC10448355; doi:10.1039/d3na00266g)
Supplement: NA-005-D3NA00266G-s001 [file NA-005-D3NA00266G-s001.pdf]

Electronic Supplementary Material (ESI) for Nanoscale Advances.
This journal is © The Royal Society of Chemistry 2023

Size Dependence of the Surface Spin Disorder and Surface Anisotropy Constant in Ferrite Nanoparticles

Marianna Gerina,[a] Marco Sanna Angotzi,[b] Valentina Mameli,[b] Veronika Gajdošová,[c] Daniel N. Rainer,[d] Milan Dopita,[e] Nina-Juliane Steinke,[f] David Filipe Coelho de Almeida Aurélio,[e] Jana Vejpravová,[e] and Dominika Zákutná*[a]

X-ray diffraction

Using PXRD, we confirmed that all samples consist of the pure spinel phase (Fd$\bar{3}$m) without the presence of other structural phases. Using the Rietveld analysis with implemented spherical harmonic function[1] (**Figure S 1**), the crystallite NP sizes of 3.1(1) (S3 sample), 6.3(1) (S6 sample), and 8.6(1) nm (S9 sample) were extracted. Moreover, the crystallite shape (insets of **Figure S 1**) shows that the coherent domain size is close to the ideal spherical shape. The lattice parameters values of prepared samples are close to the reported $CoFe_2O_4$ value (a = 8.3919 Å, PDF2 no.00-022-1086), except for the S9 sample, which shows a smaller lattice parameter as expected for NPs. S3 and S6 samples have slightly larger lattice parameters indicating possible structural disorder within the NP core.

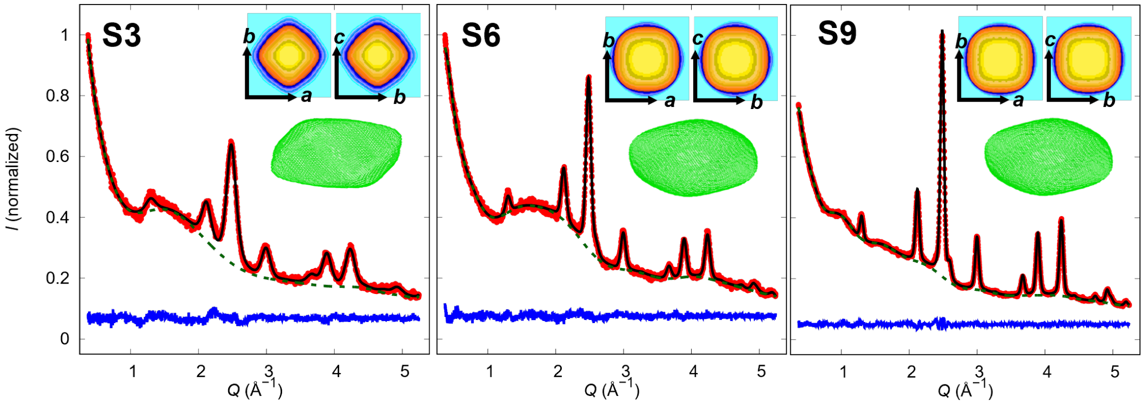

Figure S 1 Rietveld analysis of prepared samples, where red points, black, blue and dashed dark-green lines correspond to the experimental data, fit, residual, and background, respectively. The shape of the coherent domain sizes in two directions are presented as insets of the PXRD patterns.

[a] *Department of Inorganic Chemistry, Faculty of Science, Charles University, Hlavova 2030/8, 12843 Prague 2, Czech Republic.*
[b] *Department of Chemical and Geological Sciences, University of Cagliari, S.S. 554 bivio per Sestu, 09042 8 Monserrato (CA), Italy.*
[c] *Institute of Macromolecular Chemistry, Academy of Sciences of the Czech Republic, 162 06 Prague 6, Czech Republic.*
[d] *Department of Physical and Macromolecular Chemistry, Faculty of Science, Charles University, Hlavova 2030/8, 12843 Prague 2, Czech Republic.*
[e] *Department of Condensed Matter Physics, Faculty of Mathematics and Physics, Charles University, Ke Karlovu 5, 121 16 Prague 2, Czech Republic.*
[f] *Institut Laue-Langevin, 71 Avenue des Martyrs, F-38042 Grenoble, France.*
* corresponding author, E-mail: zakutnad@natur.cuni.cz

Parameter	S3	S6	S9
a (Å)	8.403(1)	8.395(1)	8.387(1)
u (x, y, z)	0.2583(3)	0.2555(2)	0.2545(1)
**profile**	Thompson-Cox-Hastings pseudo-Voigt		
zero (0.01°)	-0.067(8)	-0.002(4)	0.002(1)
BOV (Å^{-2})	4.15(5)	5.3(1)	4.08(1)
Gaus (0.01°)	2.93(5)	0.91(1)	0.44(1)
**spherical harmonics**	Laue class m3m		
K_{00}	16.0(7)	6.5(3)	5.3(1)
K_{41}	-4.2(5)	0.2(2)	0.3(1)
K_{61}	1.1(4)	-0.8(2)	0.1(1)
K_{62}	0	0	0
K_{81}	-1.7(2)	-0.2(1)	0.3(1)
R_{p} (%)	2.0	1.7	1.2
R_{exp} (%)	2.3	2.1	1.4
R_{B} (%)	1.7	2.3	0.9
R_{f} (%)	1.5	2.0	0.6
χ^2	1.3	1.1	1.3
**background**	Chebyshev polynom		
n	15	14	24
n_{total}	25	28	35

Table S 1 Summarized results from Rietveld refinement. Parameters a, u, zero, BOV, Gaus, K_{lm}, n, and n_{total} correspond to the lattice parameter, fraction position of oxygen, zero-shift, overall isotropic displacement, Gaussian broadening, and real spherical harmonics for Lorentzian size broadening, number of the background coefficients, and total number of refined parameters, respectively.

TEM/HRSTEM/SAXS/SANS analysis

The physical size and morphology of the synthesized ferrite NPs were investigated at the local and global scale utilizing TEM and SAXS/SANS analysis, respectively. The results from the lognormal distribution function from TEM micrographs analysis (**Figure S2**) are summarized in **Table S2**. HRSTEM micrographs presented in **Figure S3** reveal a tiny amorphouslayer at the nanoparticle surface, changing with nanoparticle size. Detailed micrographs show that the S3 sample is fully crystalline without the significant presence of a disordered surface, while in S6 and especially the S9 sample, a tiny non-crystalline part at the surface of nanoparticles is visible. SAXS data were best described by the simple spherical form factor, where the results from fits are shown in **Table S2**. In the case of nuclear SANS scattering cross-section, the data were ascribed with core-surfactant form factor prefixed structural information received from SAXS analyses, and only the oleic acid ligand shell thickness (d_{OA}) was refined (**Table S2**).

Parameter	S3	S6	S9
d_{XRD} (nm)	3.1(1)	6.3(2)	8.6(1)
d_{SAXS} (nm)	4.4(1)	8.1(1)	11.0(1)
σ_{SAXS} (%)	19	14	13
d_{OA} (nm)	1.30(1)	1.30(1)	1.36(2)
d_{TEM} (nm)	3.5(1)	8.1(1)	11.6(1)
σ_{TEM} (%)	14	14	11
w_{OA} (%)	54	30	14

Table S 2 Summarized results from XRD, TEM, SAXS, and nuclear SANS analysis, where d_{XRD}, d_{SAXS}, σ_{SAXS}, d_{OA}, d_{TEM}, σ_{TEM}, and ϕ_{OA} correspond to the coherent domain size from PXRD, the nuclear particle size from SAXS, the size distribution obtained from SAXS, thickness of OA shell obtained from nuclear SANS, particle diameter from TEM, size distribution from TEM, and organic mass percentage inside of samples, respectively.

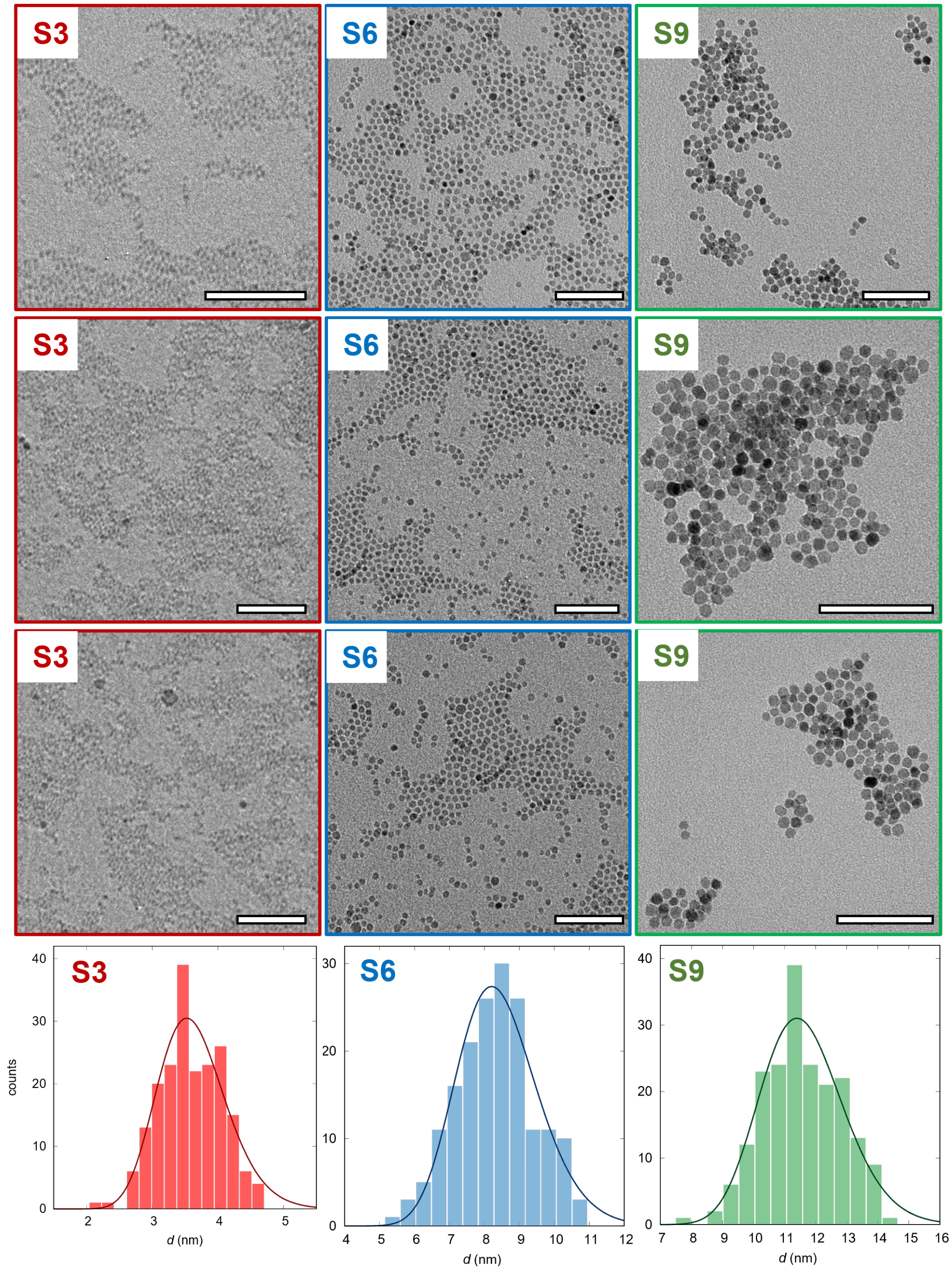

Figure S 2 TEM micrographs of S3, S6 and S9 samples with histogram of their size distribution. The full line corresponds to the fit of log-normal distribution function. Scale bars: 100 nm

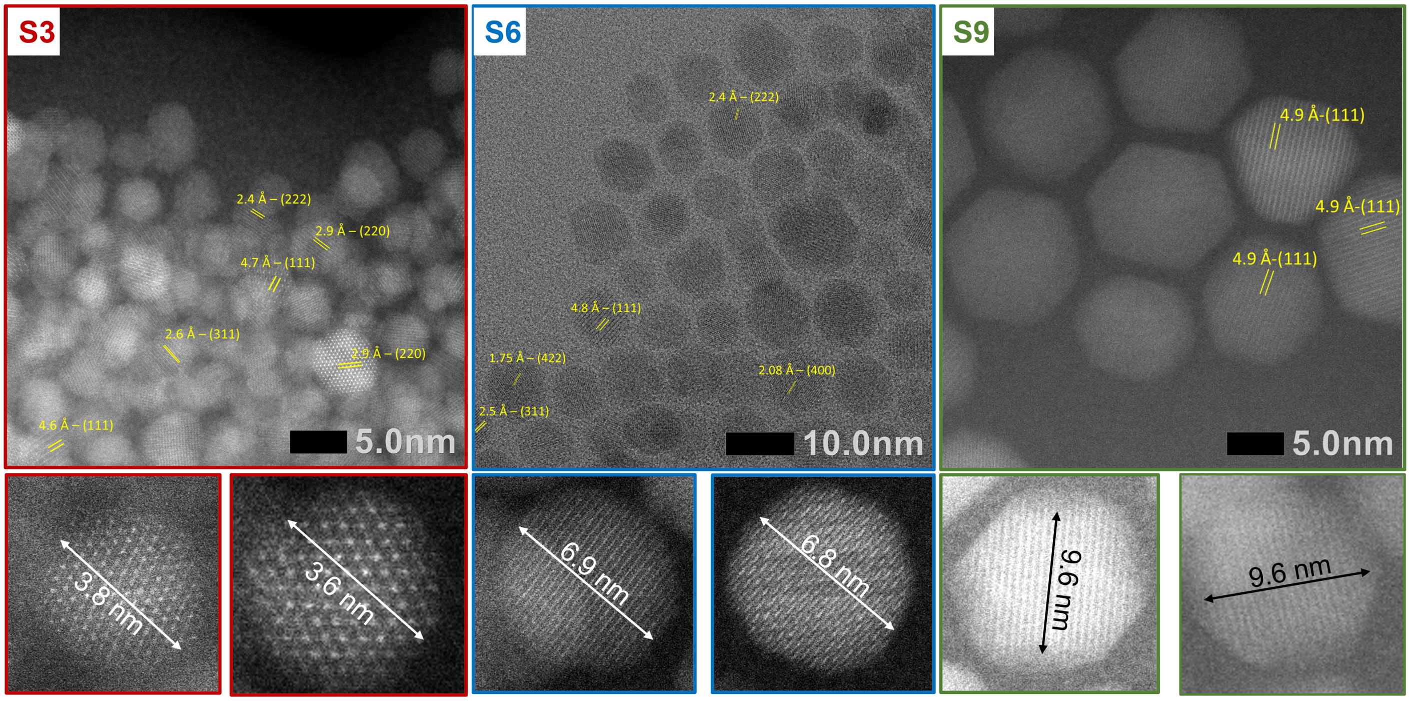

Figure S 3 upper row: HRSTEM micrographs of S3 (ADF), S6 (ABF) and S9 (ADF) samples with marked interplanar spacings (yellow) and bottom row: detailed ADF micrographs on selected nanoparticles with indication of crystalline size.

SANSPOL results

Half-polarized SANS data were fitted according to the core-shell-surfactant spherical form factor using the magnetic scattering length densities (ρ_{mag}) and disorder shell thickness (d_{dis}) as the only free parameters. All remaining parameters were fixed as obtained from SAXS and nuclear SANS analyses. The results for S3, S6, and S9 samples are summarized in **Table S3**, **Table S4**, and **Table S5**, respectively.

B (T)	d_{dis} (Å)	ρ_{mag} (10^{-6} Å^{-2})	M_z (kA/m)	$\langle M \rangle$ (kA/m)
0.01969	5(3)	0.08(4)	28(13)	15(10)
0.0298	5(2)	0.20(4)	65(14)	33(12)
0.0532	4.8(8)	0.40(4)	136(14)	66(11)
0.1038	1.0(4)	0.60(3)	204(9)	179(13)
0.677	0.7(3)	0.83(3)	285(9)	260(13)
1.336	0.6(2)	1.03(2)	354(8)	327(12)

Table S 3 SANSPOL results of S3 sample at different applied magnetic fields, B, with the magnetic scattering length density ρ_{mag}, the disorder shell thickness d_{dis}, longitudinal magnetization M_z, and the volume-averaged magnetization $\langle M \rangle$.

B (T)	d_{dis} (Å)	ρ_{mag} (10^{-6} Å^{-2})	M_z (kA/m)	$\langle M \rangle$ (kA/m)
0.00945	8(3)	0.26(5)	89(18)	44(14)
0.01969	8(1)	0.40(6)	138(22)	73(15)
0.0298	8(1)	0.60(5)	205(16)	109(13)
0.0532	4.9(8)	0.74(4)	253(13)	172(14)
0.1038	4.3(5)	1.16(3)	363(12)	258(14)
0.677	2.2(4)	1.23(3)	424(3)	359(13)
1.336	1.8(4)	1.23(3)	423(3)	368(13)

Table S 4 SANSPOL results of S6 sample at different applied magnetic fields, B, with the magnetic scattering length density ρ_{mag}, the disorder shell thickness d_{dis}, longitudinal magnetization M_z, and the volume-averaged magnetization $\langle M \rangle$.

B (T)	d_{dis} (Å)	ρ_{mag} (10^{-6} Å^{-2})	M_z (kA/m)	$\langle M \rangle$ (kA/m)
0.0298	8.7(9)	0.95(4)	328(15)	196(14)
0.0532	7.1(6)	1.06(4)	365(12)	241(12)
0.1038	6.7(5)	1.20(4)	412(11)	278(12)
0.677	5.6(4)	1.23(3)	422(10)	305(12)
1.336	5.2(4)	1.25(3)	429(10)	318(12)

Table S 5 SANSPOL results of S9 sample at different applied magnetic fields, B, with the magnetic scattering length density ρ_{mag}, the disorder shell thickness d_{dis}, longitudinal magnetization M_z, and the volume-averaged magnetization $\langle M \rangle$.

Macromagnetic properties

From the ZFC and FC curves of all prepared samples, the shift of the temperature of ZFC maxima and T_{max} with increasing particle size is observed (**Figure S4**). The blocking temperature, T_B, was obtained from the Gaussian function fit of the first derivative of the difference between FC and ZFC curves (**Table S6**). Below T_B, NPs are in the block state with ferrimagnetic ordering, and above T_B, NPs are in a superparamagnetic state. Isothermal magnetization measurements (at 4 K and 298 K) also support these observations. At 4 K, all samples have an open hysteresis with a large coercive field, while at room temperature samples, S3 and S6 have S-shape curves typical for Langevin behavior. Only the S9 sample has an open hysteresis indicating that NPs are still in the blocked state. All determined values from magnetization measurements are summarized in **Table S6**. A clear trend with increasing NPs size is also observed for coercive fields at 4 K and spontaneous magnetization at 4 and 298 K, M_s, obtained as a linear extrapolation of magnetization at high magnetic fields.

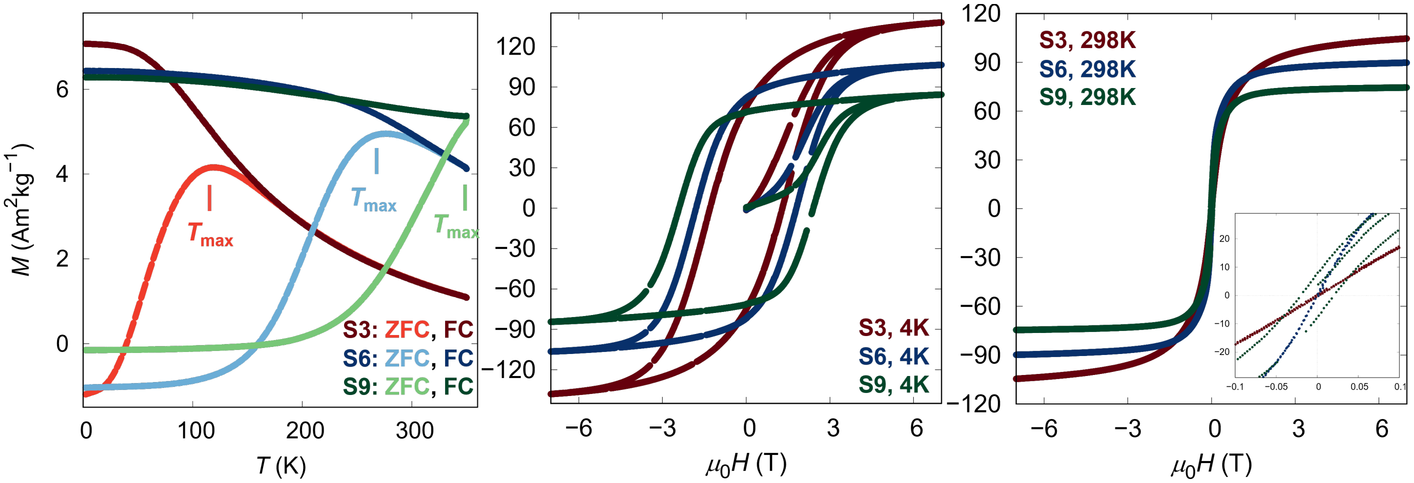

Figure S 4 (*left*) ZFC (bright points) and FC (dark points) measurements at 10 mT aplied magnetic field. The vertical lines represent the maxima of ZFC curve, T_{max}. (*middle*) Isothermal magnetization measurements at 4 K and (*right*) 298 K for all prepared samples.

Parameter	S3	S6	S9
T_{max} (K)	118(2)	270(2)	≈ 350
T_B (K)	69(1)	206(2)	319(4)
$\mu_0 H_c^{4K}$ (T)	1.28(1)	1.78(1)	2.40(1)
$\mu_0 H_c^{298K}$ (mT)	-	-	19.5(1)
M_s^{4K} (Am^{2}kg^{-1})	122.3(1)	96.9(1)	73.4(2)
M_s^{298K} (Am^{2}kg^{-1})	95.0(1)	86.2(1)	72.6(1)

Table S 6 Determined parameters from macroscopic magnetization measurements. Where T_{max} is maxima of ZFC curve, T_B the blocking temperature, $\mu_0(H_c^{4K})$ coercive field at 4 K, $\mu_0(H_c^{298K})$ coercive field at 298 K, M_s^{4K} spontaneous magnetization at 4 K, and M_s^{298K} spontaneous magnetization at 298 K.

We determine the magnetic moment distribution using the model-independent non-regularized inversion method based on a non-negative least-squares procedure (NNLS) by means using the MINORIM software[2]. **Figure S5** shows the refine-

ment of isothermal magnetizations in the SPM state and obtained M_S-weighted dipole moment distribution by applying the diamagnetic correction of $5{\cdot}10^6$ A/m.

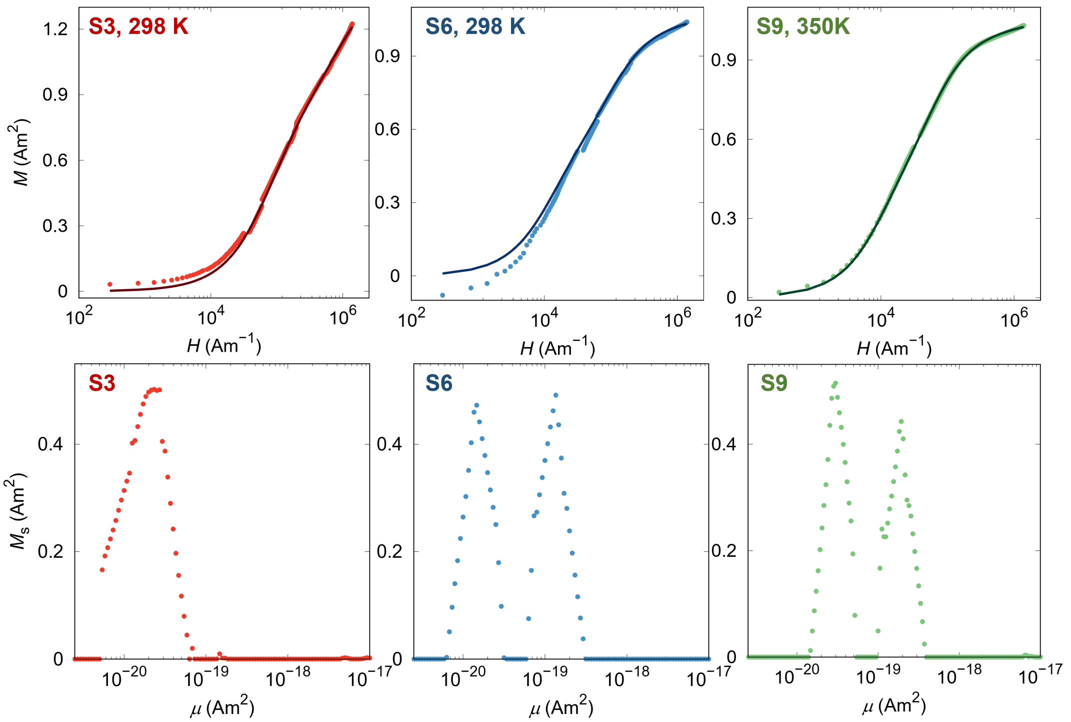

Figure S 5 (*top*) Dependence of the disorder energy and (*bottom*) probability distributions of the magnetic moment. The black and colored dark lines represent the number weighted and high evidence probability of the magnetic moment, respectively.

Micromagnetic simulations

To support the experimental results of the isothermal magnetic hysteresis loops measurements of our NPs, micromagnetic simulations were made. These were performed by using the parallel GPU mumax3 finite difference numerical code[3]. In this simulation software, the magnetization dynamics is computed by solving the following Landau–Lifshitz–Gilbert (LLG) equation

$$\frac{1}{\gamma}\frac{d\mathbf{m}}{d\mathbf{t}} = -\frac{1}{1+\alpha^2}(\mathbf{m}\times\mathbf{H}_{\mathrm{eff}}) - \frac{\alpha}{1+\alpha^2}\mathbf{m}\times(\mathbf{m}\times\mathbf{H}_{\mathrm{eff}}) \tag{1}$$

where, γ is the gyromagnetic ratio, $\mathbf{m} = \mathbf{M}/M_S$ is the normalized magnetization vector, α is the Gilbert damping parameter and $\mathbf{H}_{\mathrm{eff}}$ is the total effective field. The $\mathbf{H}_{\mathrm{eff}}$ includes the typical interactions due to exchange, anisotropy, demagnetization and external fields, as well as a thermal field interaction term.

The simulations were based on a single NP, composed of a mesh of several cubic computational cells of 1 nm side. Both uniaxial and cubic anisotropies were taken into account in separate simulations. This is since CoFe NPs can present a behavior more consistent with the uniaxial anisotropy crystal structure, even though bulk CoFe is known to have cubic anisotropy. **Table S 7** shows the material parameters used in the simulations, which include the uniaxial (K_u) and cubic (K_c) anisotropy constants, their corresponding easy axis vector directions, saturation magnetization M_S, exchange energy A_{ex}, as well as damping parameter α used. Note that the units of these quantities have to be converted to the ones shown in the table since it is the ones mumax3 takes for the simulations.

**Sample**	M_S (Am^{-1})	A_{ex} (Jm^{-1})	K_u (Jm^{-3})	K_c(Jm^{-3})	α
**S3**	$6.31{\cdot}10^5$	$1.5{\cdot}10^{-11}$	$5{\cdot}10^0$ @ (1,0,0)	$3.5{\cdot}10^3$ @ (1,0,0);(1,1,0)	0.01
**S6**	$6.31{\cdot}10^5$	$1.5{\cdot}10^{-11}$	$5{\cdot}10^4$ @ (1,0,0)	$1.0{\cdot}10^2$ @ (1,0,0);(1,1,0)	0.01
**S9**	$6.31{\cdot}10^5$	$1.5{\cdot}10^{-11}$	$5{\cdot}10^5$ @ (1,0,0)	$5.0{\cdot}10^2$ @ (1,0,0);(1,1,0)	0.01

Table S 7 Parameters used in mumax3 for the hysteresis loop simulations of samples S3, S6 and S9.

As for the external magnetic field, the simulations were performed by sweeping the field from -7 T to 7 T and back again in 0.1 T increments, each for $1.5 \cdot 10^{-11}$ s of simulation run time. The magnetic field direction is also applied slightly off the easy axis of the NP (0.4 degrees) to avoid "computational pinning" of the magnetization. The thermal field[3] contribution to the effective field was determined by assuming a 4 K temperature for the different simulations.

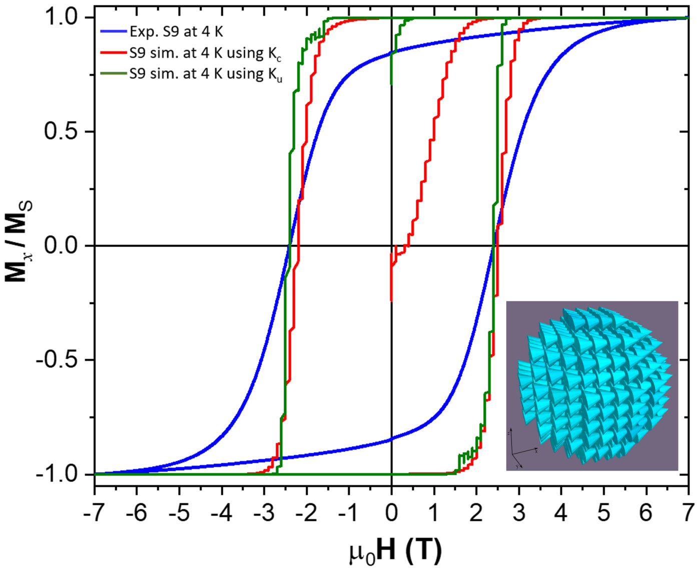

Figure S 6 Experimental hysteresis loop for sample S9 (Blue). Single 12 nm diameter NP simulation under a sweeping external magnetic field, characterized by the uniaxial anisotropy with easy direction (1,0,0) (Green) and characterized by the cubic anisotropy with easy directions along (1,0,0), (1,1,0), in decreasing strength (Red). Inset: Simulated NP showing magnetization direction of individual spins.

Figure S6 shows the results of the simulated S9 NP, where we can see that both the uniaxial and cubic anisotropy simulations can represent the experimental results fairly well of the isothermal hysteresis loops. The main difference is in the squareness of the hysteresis loop, which can be explained by the fact that we simulate only one NP with a well-defined anisotropy easy axis direction, whereas the experimental sample is composed by an extremely large number of particles with random anisotropy directions when measured. As for the coercivity, the simulations also show very similar results, however, to achieve this, it was necessary to define a smaller anisotropy constant than that measured for the S9 sample. Moreover, in the case of the simulation with cubic anisotropy, the constant had to be much smaller than that of S9, pointing to the uniaxial anisotropy as a better representation of the magnetization dynamics of such small NPs.

Very similar results are found for the simulations of single NP of samples S6 (**Figure S7**) and S3 (**Figure S8**). However, in the case of sample S3 the simulation using the cubic anisotropy is now the one with the anisotropy constant closer to that of the experimental S3 sample. Still, all the anisotropies reached in the simulations to match the coercivity seen for the experimental samples are different. This could be due to several different factors related to the limitations of the simulation model like; impossible to simulate all the particles present in the sample, and thus we simulate just one to have an idea of the general behavior; the size of the simulated NP is slightly different from the average size of the particles in the sample due to model restrictions; the material parameters of a single NP are probably different from that of the ensemble of the particles in the sample.

Nevertheless, the simulations overall show a very good agreement between the hysteresis loop shape and coercivity seen for the experimental samples. This means the effective field interactions used in the micromagnetic model, although not perfect, are a good representation of the magnetization dynamics involved.

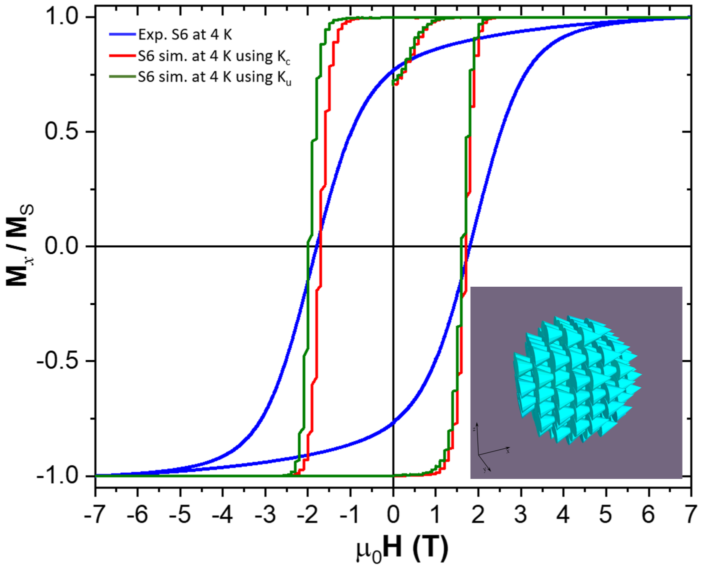

Figure S 7 Experimental hysteresis loop for sample S6 (Blue). Single 8 nm diameter NP simulation under a sweeping external magnetic field, characterized by the uniaxial anisotropy with easy direction (1,0,0) (Green) and characterized by the cubic anisotropy with easy directions along (1,0,0), (1,1,0), in decreasing strength (Red). Inset: Simulated NP showing magnetization direction of individual spins.

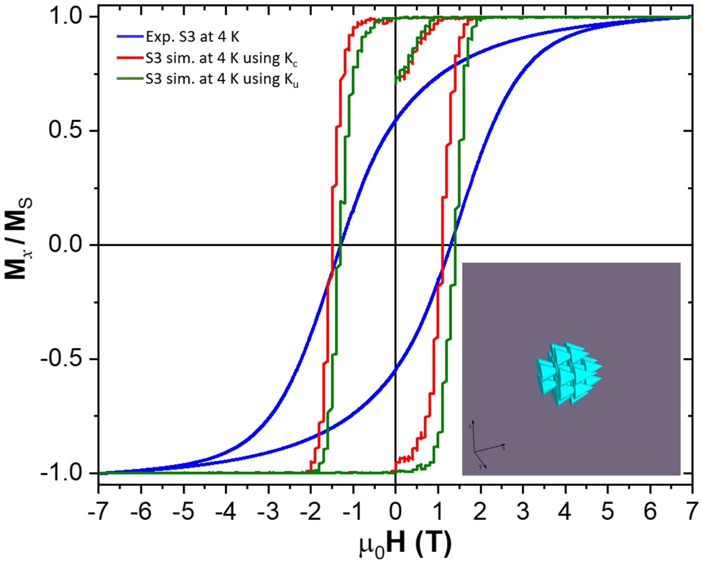

Figure S 8 Experimental hysteresis loop for sample S9 (Blue). Single 4 nm diameter NP simulation under a sweeping external magnetic field, characterized by the uniaxial anisotropy with easy direction (1,0,0) (Green) and characterized by the cubic anisotropy with easy directions along (1,0,0), (1,1,0), in decreasing strength (Red). Inset: Simulated NP showing magnetization direction of individual spins.

Notes and references

1 J. Bergmann, T. Monecke and R. Kleeberg, *Journal of Applied Crystallography*, 2001, **34**, 16–19.
2 J. van Rijssel, B. W. Kuipers and B. H. Erné, *Journal of Magnetism and Magnetic Materials*, 2014, **353**, 110–115.
3 A. Vansteenkiste, J. Leliaert, M. Dvornik, M. Helsen, F. Garcia-Sanchez and B. Van Waeyenberge, *AIP Advances*, 2014, **4**, 107133.
